# Recommendation of premarital genetic screening in the Syrian Jewish community based on mutation carrier frequencies within Syrian Jewish cohorts

**DOI:** 10.1002/mgg3.1756

**Published:** 2021-07-20

**Authors:** David A. Zeevi, Wendy K. Chung, Chaim Levi, Sholem Y. Scher, Rachel Bringer, Yael Kahan, Hagit Muallem, Rinat Benel, Yoel Hirsch, Tzvi Weiden, Ahron Ekstein, Josef Ekstein

**Affiliations:** ^1^ Dor Yeshorim The Committee for Prevention of Jewish Genetic Diseases Jerusalem Israel; ^2^ Columbia University New York NY USA; ^3^ Dor Yeshorim The Committee for Prevention of Jewish Genetic Diseases Brooklyn NY USA

**Keywords:** carrier frequency, Iranian Jewish, premarital genetic screening, Syrian Jewish

## Abstract

**Background:**

There is a paucity of information available regarding the carrier frequency for autosomal recessive pathogenic variants among Syrian Jews. This report provides data to support carrier screening for a group of autosomal recessive conditions among Syrian Jews based on the population frequency of 40 different pathogenic variants in a cohort of over 3800 individuals with Syrian Jewish ancestry.

**Methods:**

High throughput PCR amplicon sequencing was used to genotype 40 disease‐causing variants in 3840 and 5279 individuals of Syrian and Iranian Jewish ancestry, respectively. These data were compared with Ashkenazi Jewish carrier frequencies for the same variants, based on roughly 370,000 Ashkenazi Jewish individuals in the Dor Yeshorim database.

**Results:**

Carrier screening identified pathogenic variants shared among Syrian, Iranian, and Ashkenazi Jewish groups. In addition, alleles unique to each group were identified. Importantly, 8.2% of 3401 individuals of mixed Syrian Jewish ancestry were carriers for at least one pathogenic variant.

**Conclusion:**

The findings of this study support the clinical usefulness of premarital genetic screening for individuals with Syrian Jewish ancestry to reduce the incidence of autosomal recessive disease among persons with Syrian Jewish heritage.

## Graphical abstract

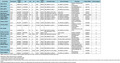

## 1 INTRODUCTION

Over the past few decades, premarital genetic testing has served as a model preventative measure to prevent the birth of babies affected with serious medical conditions in Jewish communities. Premarital carrier screening programs, such as that in Dor Yeshorim, have contributed to the dramatic reduction of recessive disease incidence in Ashkenazi Jews, especially for Tay Sachs and cystic fibrosis (Ekstein & Katzenstein, 2001; Kornreich et al., 2004). In Israel, preconception/prenatal carrier screening has been offered to Jewish individuals since 1978 (Zlotogora, 2014). Beginning with Tay Sachs screening, carrier screening has expanded to tens and hundreds of disease‐causing alleles for autosomal recessive conditions in Jewish populations around the world (Zlotogora, 2019). In the US, the American College of Obstetricians and Gynecologists (ACOG) established guidelines for genetic testing of Ashkenazi Jewish individuals in 2008 (“Committee Opinion No. 691,” 2017). However, although various screening programs have made efforts to reach other non‐Ashkenazi Jewish groups, carrier screening is utilized less frequently in non‐Ashkenazi Jewish communities (Akler et al., 2020; Bloch, 2009).

Intramarriage within parts of the Ashkenazi Jewish population are well documented (Carmi et al., 2014) and is a contributor to a high carrier frequency for certain disease‐causing variants for recessive conditions in this ancestral group (Zlotogora, 2014). However, intramarriage is not unique to Ashkenazi Jewry. To varying extents, Sephardi and Mizrahi Jewish groups have a history of intramarriage, unique to their geographical origin (Goldschmidt et al., 1960). These population characteristics have led the Israeli Society of Medical Geneticists to establish carrier screening guidelines for all Jews based on country of origin, indicating that all Jewish groups carry pathogenic disease‐causing recessive variants and that screening should be tailored based upon genetic ancestry (Zlotogora, 2019).

Syrian and Iranian Jews are genetically quite similar (Atzmon et al., 2010), yet unlike disease‐causing variants common to Iranian Jews (Dagan & Gershoni‐Baruch, 2010), much information is lacking regarding the frequency of pathogenic variants in Syrian Jews. Hence, we chose Iranian Jews as a comparison group for the assessment of allele frequencies within the Syrian Jewish population. In this study, we characterize the frequency of several pathogenic variants within Syrian and Iranian Jewish cohorts. We establish ancestry‐specific frequencies of certain variants in each group, and we describe some variants shared between Syrians and Iranians and between Syrians and Ashkenazis. The latter findings suggest that it may be most practical to apply pan‐Jewish variant screening for effective carrier screening within the Syrian Jewish community.

## 2 MATERIALS AND METHODS

### 2.1 Ethnic origin definition of study participants

Participants in the screening program provided information regarding the ancestry of all four grandparents. Self‐identified ancestry has been previously shown to be accurate (Need et al., 2009). For inclusion in the study, individuals with at least one maternal or one paternal Syrian/Iranian grandparent were selected. For families with 2 or more siblings, 1 sibling was randomly selected for analysis so that the final cohort would include only unrelated Syrian/Iranian individuals. Altogether, 439 unrelated 100% Syrian (with 4 Syrian grandparents), 3401 unrelated mixed Syrian (with less than 4 Syrian grandparents), and 5279 unrelated mixed Iranians were included in the analysis. For comparison, carrier frequencies in Ashkenazi Jews were calculated from roughly 370,000 Ashkenazi Jewish samples in the Dor Yeshorim database.

### 2.2 Genotyping of sequence variants and the FRDA expansion with high throughput amplicon sequencing

A multiplex PCR assay was used to target the single nucleotide variants (SNVs)/small insertions/small deletions in Tables 1 and 2, as well as the Friedrich's ataxia (FRDA) GAA expansion locus in intron 1 of the *FXN* gene. Multiplex PCR products from each sample were barcode indexed before sequencing on an Illumina MiSeq. Resultant sequencing reads were aligned to the reference genome (hg38) and relevant variants were genotyped using GATK UnifiedGenotyper/HaplotypeCaller packages (Broad Institute). Genotyping assays for each SNV/small insertion/small deletion were validated by testing at least 2 or 3 different heterozygote control samples. For FRDA expansion carrier detection, the following extra analytical steps were performed. Sequencing reads were aligned to an “STRdecoys.fasta” reference (obtained from [Dashnow et al., 2018]) which was supplemented with the expected non‐GAA *FXN* intron 1 amplicon sequence from hg38. Using this mapping strategy, it was expected that FRDA repeat‐expanded reads would map to both the decoy GAA “chromosome” and the *FXN* intron 1 “chromosome.” These split‐reads were extracted by samblaster (Faust & Hall, 2014) followed by counting the total number of GAA repeats per sample and GAA repeat‐bearing reads per sample. Furthermore, within repeat‐bearing reads, the number of GAA repeats per read was calculated for each sample. After sorting the number of repeats per read, the 3 highest repeat‐per‐read frequencies per sample were saved to a final report. True FRDA carriers were detected when the top 2 repeat‐per‐read frequencies (RPRF) all exceeded 35 GAA repeats. Otherwise, all samples with less than 2 RPRF >35 GAA repeats were reported as non‐carriers. All positive FRDA custom amplicon sequencing‐based results were validated by an established fluorescent repeat‐primed PCR‐based method (Ciotti et al., 2004). In addition, the specificity of the sequencing‐based method was determined by confirming 367 random non‐carrier samples (as determined by high throughput sequencing) using the fluorescent PCR assay.

**TABLE 1:** Disease‐causing variants for which carrier state was identified in at least one Syrian Jewish individual with 4 Syrian Jewish grandparents at Dor Yeshorim

Variant name	Chromosome	Position (hg38)	rsID	Ref	Alt	Gene	OMIM Gene	Nucleotide change^a^	Amino acid change^b^	Phenotype	Syrian Jewish carrier frequency (no. carriers/*n*)	Syrian Jewish carrier frequency %	Ashkenazi Jewish carrier frequency (no. carriers/*n*)	Ashkenazi Jewish carrier frequency %	Followed up in Table 2
ABCC8:c.3989‐9G>A	chr11	17397055	rs151344623	C	T	ABCC8	600509	NM_000352.6:c.3989‐9G>A	splice acceptor	HYPERINSULINEMIC HYPOGLYCEMIA, FAMILIAL, 1	1/339	0.29	3969/235602	1.68	NO
AGXT:c.731T>C	chr2	240875159	rs121908525	T	C	AGXT	604285	NM_000030.3:c.731T>C	NP_000021.1:p.Ile244Thr	PRIMARY HYPEROXALURIA TYPE 1	4/369	1.08	0/21050	0.00	YES
ARSA:c.449C>T	chr22	50627182	rs199476375	G	A	ARSA	607574	NM_000487.6:c.449C>T	NP_000478.3:p.Pro150Leu	METACHROMATIC LEUKODYSTROPHY	5/376	1.33	1/51587	0.00	YES
ARSA:c.854+3A>G	chr22	50626588	rs1057524566	T	C	ARSA	607574	NM_000487.6:c.854+3A>G	splice donor	METACHROMATIC LEUKODYSTROPHY	5/376	1.33	0/46388	0.00	YES
BLM:c.2208T>G	chr15	90766924	rs865899765	T	G	BLM	604610	NM_000057.4:c.2208T>G	NP_000048.1:p.Tyr736Ter	BLOOM SYNDROME	1/404	0.25	3265/335777	0.97	NO
CFTR:c.3846G>A	chr7	117642566	rs77010898	G	A	CFTR	602421	NM_000492.4:c.3846G>A	NP_000483.3:p.Trp1282Ter	CYSTIC FIBROSIS	3/435	0.69	6721/335635	2.00	NO
CFTR:c.254G>A	chr7	117509123	rs75961395	G	A	CFTR	602421	NM_000492.4:c.254G>A	NP_000483.3:p.Gly85Glu	CYSTIC FIBROSIS	2/381	0.52	0/97562	0.00	YES
CFTR:c.1624G>T	chr7	117587778	rs113993959	G	T	CFTR	602421	NM_000492.4:c.1624G>T	NP_000483.3:p.Gly542Ter	CYSTIC FIBROSIS	2/435	0.46	737/335635	0.22	NO
CFTR:c.1521_1523del	chr7	117559590	rs113993960	ATCT	A	CFTR	602421	NM_000492.4:c.1521_1523del	NP_000483.3:p.Phe508del	CYSTIC FIBROSIS	2/438	0.46	3965/335635	1.18	YES
CFTR:c.2989‐1G>A	chr7	117610518	rs397508470	G	A	CFTR	602421	NM_000492.4:c.2989‐1G>A	splice acceptor	CYSTIC FIBROSIS	1/329	0.30	0/223335	0.00	NO
CNGB3:c.467C>T	chr8	86670970	rs139207764	G	A	CNGB3	605080	NM_019098.4:c.467C>T	NP_061971.3:p.Ser156Phe	ACHROMATOPSIA	7/376	1.86	26/44392	0.06	YES
COL6A2:c.1402C>T	chr21	46121067	rs374669775	C	T	COL6A2	120240	NM_001849.3:c.1402C>T	NP_001840.3:p.Arg468Ter	ULLRICH CONGENITAL MUSCULAR DYSTROPHY TYPE 1	13/331	3.93	0/5922	0.00	YES
CYP11B1:c.992C>T	chr8	142875841	rs1326688256	G	A	CYP11B1	610613	NM_000497.3:c.992C>T	NP_000488.3:p.Ala331Val	CONGENITAL ADRENAL HYPERPLASIA	10/376	2.66	0/44988	0.00	YES
DHCR7:c.964‐1G>C	chr11	71435840	rs138659167	C	G	DHCR7	602858	NM_001360.3:c.964‐1G>C	splice acceptor	SMITH‐LEMLI‐OPITZ SYNDROME	2/227	0.88	2016/88773	2.27	NO
DSE:c.387delC	chr6	116399636	N/A	AC	A	DSE	605942	NM_013352.4:c.387delC	NP_037484.1:p.Tyr129Ter	EHLERS‐DANLOS SYNDROME, MUSCULOCONTRACTURAL TYPE2	1/369	0.27	0/21009	0.00	YES
ESCO2:c.1674‐2A>G	chr8	27803304	rs80359869	A	G	ESCO2	609353	NM_001017420.3:c.1674‐2A>G	splice acceptor	ROBERTS SYNDROME	2/376	0.53	0/51519	0.00	YES
FAM161A:c.1567C>T	chr2	61839437	rs202193201	G	A	FAM161A	613596	NM_001201543.2:c.1567C>T	NP_001188472.1:p.Arg523Ter	RETINITIS PIGMENTOSA 28	3/154	1.95	1/49492	0.00	NO
FXN:GAA expansion	chr9	69037287	N/A	GAA	GAA>>	FXN	606829	NM_000144:GAA expansion	N/A	FRIEDREICH ATAXIA	3/331	0.91	14/5109	0.27	YES
G6PC:c.247C>T	chr17	42903947	rs1801175	C	T	G6PC1	613742	NM_000151.4:c.247C>T	NP_000142.2:p.Arg83Cys	GLYCOGEN STORAGE DISEASE TYPE 1A	2/397	0.50	4816/335799	1.43	NO
GBA:c.1448T>C	chr1	155235252	rs421016	A	G	GBA	606463	NM_000157.4:c.1448T>C	NP_000148.2:p.Leu483Pro	GAUCHER DISEASE	1/101	0.99	88/72333	0.12	NO
GJB2:c.167del	chr13	20189414	rs80338942	CA	C	GJB2	121011	NM_004004.6:c.167del	NP_003995.2:p. Leu56fs	NONSYNDROMIC DEAFNESS	8/368	2.17	929/32870	2.83	YES
GJB2:c.269T>C	chr13	20189313	rs80338945	A	G	GJB2	121011	NM_004004.6:c.269T>C	NP_003995.2:p.Leu90Pro	NONSYNDROMIC DEAFNESS	1/104	0.96	7/32171	0.02	NO
GNE:c.2228T>C	chr9	36217399	rs28937594	A	G	GNE	603824	NM_001128227.3:c.2228T>C	NP_001121699.1:p.Met743Thr	INCLUSION BODY MYOPATHY (HIBM)	8/376	2.13	1/51647	0.00	YES
MLC1:c.176G>A	chr22	50084727	rs80358242	C	T	MLC1	605908	NM_015166.3:c.176G>A	NP_055981.1:p.Gly59Glu	MEGALENCEPHALIC LEUKOENCEPHALOPATHY WITH SUBCORTICAL CYSTS 1	1/273	0.37	0/53648	0.00	NO
MMACHC:c.271dup	chr1	45507544	rs398124292	T	TA	MMACHC	609831	NM_015506.3:c.271dup	NP_056321.2:p.Arg91fs	METHYLMALONIC ACIDURIA AND HOMOCYSTINURIA, cblC TYPE	1/154	0.65	372/49724	0.75	NO
NDUFS4:c.355G>C	chr5	53658555	rs747359752	G	C	NDUFS4	602694	NM_002495.4:c.355G>C	NP_002486.1:p.Asp119His	LEIGH SYNDROME TYPE 1	2/376	0.53	0/46275	0.00	YES
OTOF:c.5193‐1G>A	chr2	26462182	rs111033373	C	T	OTOF	603681	NM_194248.3:c.5193‐1G>A	splice acceptor	DEAFNESS, AUTOSOMAL RECESSIVE	4/101	3.96	0/30796	0.00	NO
OTOF:c.4227+1G>T	chr2	26467364	rs397515601	C	A	OTOF	603681	NM_194248.3:c.4227+1G>T	splice donor	DEAFNESS, AUTOSOMAL RECESSIVE	1/102	0.98	0/30826	0.00	NO
PEX2:c.355C>T	chr8	76983824	rs61752123	G	A	PEX2	170993	NM_000318.3:c.355C>T	NP_000309.2:p.Arg119Ter	PEROXISOME BIOGENESIS DISORDER 5A (ZELLWEGER)	1/154	0.65	379/49883	0.76	NO
SMPD1:c.1829G>A	chr11	6394540	rs140269316	G	A	SMPD1	607608	NM_000543.5:c.1829G>A	NP_000534.3:p.Arg610His	NIEMANN‐PICK DISEASE	1/93	1.08	0/25901	0.00	NO
SMPD1:c.1826_1828GCC	chr11	6394536	rs120074118	TGCC	T	SMPD1	607608	NM_000543.5:c.1826_1828GCC	NP_000534.3:p.Arg610del	NIEMANN‐PICK DISEASE	2/380	0.53	66/304382	0.02	NO
TRPM1:36.8KB DEL	chr15	31062999‐31099445	N/A	N/A	N/A	TRPM1	603576	36.8KB DEL, EX2‐7^c^	N/A	CONGENITAL STATIONARY NIGHT BLINDNESS	2/133	1.50	946/37796	2.50	NO
VAC14:c.2005G>T	chr16	70695574	rs1363536856	C	A	VAC14	604632	NM_018052.5:c.2005G>T	NP_060522.3:p.Val669Leu	STRIATONIGRAL DEGENERATION	1/367	0.27	0/30779	0.00	YES

Note

Variants are sorted alphabetically according to gene name and then by Syrian Jewish carrier frequency from highest to lowest. Carrier frequencies in individuals with 4 Ashkenazi Jewish grandparents are shown for comparison.

Abbreviations: N/A, not applicable; PMID, Pubmed ID.

^a^ Genbank transcript accession number:nucleotide change.

^b^ Genbank protein accession number:amino acid change (where relevant).

^c^ This copy number variant is described in PMID: 31645983.

**TABLE 2:** Metadata for variants assayed by high throughput sequencing in this report

Variant Name	Chromosome	Position (hg38)	rsID	Ref	Alt	Gene	OMIM Gene	Nucleotide change^a^	Amino acid change^b^	Phenotype	Citation (PMID)	Variant Category^c^
AGXT:c.731T>C	chr2	240875159	rs121908525	T	C	AGXT	604285	NM_000030.3:c.731T>C	NP_000021.1:p.Ile244Thr	PRIMARY HYPEROXALURIA TYPE 1	9192270	**1**
AIRE:c.254A>G	chr21	44286678	rs179363882	A	G	AIRE	607358	NM_000383.4:c.254A>G	NP_000374.1:p.Tyr85Cys	AUTOIMMUNE POLYENDOCRINOPATHY	10677297	**2**
ARSA:c.449C>T	chr22	50627182	rs199476375	G	A	ARSA	607574	NM_000487.6:c.449C>T	NP_000478.3:p.Pro150Leu	METACHROMATIC LEUKODYSTROPHY	10381328	**1**
ARSA:c.854+3A>G	chr22	50626588	rs1057524566	T	C	ARSA	607574	NM_000487.6:c.854+3A>G	splice donor	METACHROMATIC LEUKODYSTROPHY	1670590 ^4^	**1**
BLM:c.98+1G>T	chr15	90747491	rs750293380	G	T	BLM	604610	NM_001287246.2:c.98+1G>T	splice donor	BLOOM SYNDROME	17407155	**1**
CFTR:c.1521_1523del	chr7	117559590	rs113993960	ATCT	A	CFTR	602421	NM_000492.4:c.1521_1523del	NP_000483.3:p.Phe508del	CYSTIC FIBROSIS	2475911, 2570460	**5**
CFTR:c.254G>A	chr7	117509123	rs75961395	G	A	CFTR	602421	NM_000492.4:c.254G>A	NP_000483.3:p.Gly85Glu	CYSTIC FIBROSIS	15176679	**1**
CNGA3:c.1585G>A	chr2	98396755	rs104893619	G	A	CNGA3	600053	NM_001298.3:c.1585G>A	NP_001289.1:p.Val529Met	ACHROMATOPSIA	20549516	**4**
CNGB3:c.467C>T	chr8	86670970	rs139207764	G	A	CNGB3	605080	NM_019098.4:c.467C>T	NP_061971.3:p.Ser156Phe	ACHROMATOPSIA	15657609	**1**
COL6A2:c.1402C>T	chr21	46121067	rs374669775	C	T	COL6A2	120240	NM_001849.3:c.1402C>T	NP_001840.3:p.Arg468Ter	ULLRICH CONGENITAL MUSCULAR DYSTROPHY TYPE 1	23326386	**1**
CYP11B1:c.992C>T	chr8	142875841	rs1326688256	G	A	CYP11B1	610613	NM_000497.3:c.992C>T	NP_000488.3:p.Ala331Val	CONGENITAL ADRENAL HYPERPLASIA	8768848	**1**
DSE:c.387delC	chr6	116399636	N/A	AC	A	DSE	605942	NM_013352.4:c.387delC	NP_037484.1:p.Tyr129Ter	EHLERS‐DANLOS SYNDROME, MUSCULOCONTRACTURAL TYPE2	23704329 ^d^	**1**
ESCO2:c.1674‐2A>G	chr8	27803304	rs80359869	A	G	ESCO2	609353	NM_001017420.3:c.1674‐2A>G	splice acceptor	ROBERTS SYNDROME	16380922	**1**
FXN:GAA expansion	chr9	69037287	N/A	GAA	GAA>>	FXN	606829	NM_000144:GAA expansion	N/A	FRIEDREICH ATAXIA	8596916	**1**
GJB2:c.167del	chr13	20189414	rs80338942	CA	C	GJB2	121011	NM_004004.6:c.167del	NP_003995.2:p.Leu56fs	NONSYNDROMIC DEAFNESS	9285800	**1**
GNE:c.2228T>C	chr9	36217399	rs28937594	A	G	GNE	603824	NM_001128227.3:c.2228T>C	NP_001121699.1:p.Met743Thr	INCLUSION BODY MYOPATHY (HIBM)	11528398	**3**
GPT2:c.159C>G	chr16	46906858	rs786203999	C	G	GPT2	138210	NM_133443.4:c.459C>G	NP_597700.1:p.Ser153Arg	DEVELOPMENTAL ENCEPHALOPATHY	25758935	**1**
LIPA:c.260G>T	chr10	89228368	rs587778878	C	A	LIPA	613497	NM_000235.4:c.260G>T	NP_000226.2:p.Gly87Val	WOLMAN'S DISEASE	21291321	**2**
NDUFS4:c.355G>C	chr5	53658555	rs747359752	G	C	NDUFS4	602694	NM_002495.4:c.355G>C	NP_002486.1:p.Asp119His	LEIGH SYNDROME TYPE 1	19364667	**1**
TYMP:c.433G>A	chr22	50528595	rs121913037	C	T	TYMP	131222	NM_001953.5:c.433G>A	NP_001944.1:p.Gly145Arg	MNGIE‐MITOCHONDRIAL NEUROGASTROINTESTINAL ENCEPHALOMYOPATHY	9924029	**4**
USH2A:c.236_239dup	chr1	216422097	rs1553258097	G	GGTAC	USH2A	608400	NM_007123.5:c.236_239dup	NP_009054.5:p.Gln81fs	USHER SYNDROME TYPE 2A	10738000	**4**
VAC14:c.2005G>T	chr16	70695574	rs1363536856	C	A	VAC14	604632	NM_018052.5:c.2005G>T	NP_060522.3:p.Val669Leu	STRIATONIGRAL DEGENERATION	31387860	**1**

Note

Variants are sorted alphabetically according to gene name.

Abbreviations: N/A, not applicable; PMID, Pubmed ID.

^a^ Genbank transcript accession number:nucleotide change.

^b^ Genbank protein accession number:amino acid change.

^c^ Variant category definitions: (1) Variant identified in family of Syrian Jewish ethnicity; (2) Variant identified in family of Iranian Jewish ethnicity; (3) Variant identified in families of either Syrian or Iranian Jewish ethnicity; (4) Variants recommended for carrier screening in Iranian Jews; (5) Pan‐ethnic variant.

^d^ Reference describing bi‐allelic loss‐of‐function variant/s in the indicated gene that associate with the indicated phenotype. The given variant is novel but predicted to be loss‐of‐function.

## 3 RESULTS

### 3.1 Preliminary assessment of the frequency of disease‐causing variants in Syrian Jews

To assess carrier frequency in Syrian Jews, we genetically tested a small cohort of 438 unrelated individuals of 100% Syrian Jewish descent (all 4 grandparents of Syrian Jewish ancestry) for at least one disease‐causing variant using amplicon sequencing. Table 1 lists 33 pathogenic variants, implicated in 25 different genetic disorders, for which a pathogenic variant was identified in at least one Syrian Jewish individual with 4 Syrian grandparents at Dor Yeshorim. Variant frequencies for Ashkenazi Jews are provided for comparison. We identified relatively high Syrian Jewish carrier frequencies (>0.68%) for well‐established Ashkenazi Jewish variants with carrier frequencies over 2% in Ashkenazi Jews (nonsyndromic deafness‐associated GJB2:c.167del; congenital stationary night blindness‐associated TRPM1:36.8KB DEL; Smith‐Lemli‐Opitz‐associated DHCR7:c.964‐1G>C; and cystic fibrosis‐associated CFTR:c.3846G>A; Table 1). In addition, Syrian Jews carried variants that are less frequent in Ashkenazis and implicated in the following autosomal recessive disorders: achromatopsia, congenital adrenal hyperplasia (*CYP11B1* gene), Friedreich ataxia, Gaucher disease, inclusion body myopathy, metachromatic leukodystrophy, Niemann–Pick disease, primary hyperoxaluria type 1, retinitis pigmentosa 28, and Ullrich congenital muscular dystrophy type 1.

### 3.2 Pathogenic variant selection for in‐depth investigation of allele frequency in Syrian and Iranian Jews

To further expand the generalizability of the results in Table 1, we assembled a larger cohort of 3401 unrelated “mixed” Syrian Jewish (with at least 1 but less than 4 Syrian Jewish grandparents) and 5279 unrelated “mixed” Iranian Jewish individuals for comparison. For this analysis, a set of 22 different disease‐causing pathogenic variants (underlying 20 different diseases) were selected for further investigation (Table 2). For 18 of these variants, at least one family of Syrian and/or Iranian Jewish ancestry approached Dor Yeshorim for assistance with the genetic diagnosis of an affected child or for assistance with premarital screening of a pre‐existing disease in the family. Three other variants were selected based on Israel Ministry of Health recommendations for genetic screening of Iranian Jews; and CFTR:c.1521_1523del was included due to its established pan‐ethnic prevalence (Palomaki et al., 2004; “Worldwide Survey of the Delta F508 Mutation—Report from the Cystic Fibrosis Genetic Analysis Consortium,” 1990). We note that other disease‐causing variants were reported previously in Syrian Jews (Table S1); however, we could not include these variants in our screen because heterozygous control samples for each variant were not available to us for genotyping assay validation. Nonetheless, we summarize these 14 variants underlying 11 different conditions in Table S1 to consolidate established disease‐causing variants in Syrian Jews at the time of this publication.

Within the 22 screened variants, 18 family‐derived variants were further categorized according to the ethnicity of the affected child as “category 1” (Syrian only), “category 2” (Iranian only), or “category 3” (Syrian and Iranian) variants (Table 2). Category 4 variants were included according to Israel Ministry of Health guidelines, and the category 5 variant is the pan‐ethnic CFTR:c.1521_1523del (see Table 2 for categorical variant definitions).

### 3.3 Carrier frequency in the Syrian and Iranian Jewish populations

To determine the frequency of carriers for the variants in Table 2, mixed Syrian, mixed Iranian, and mixed Ashkenazi Jewish individuals were screened using high throughput amplicon sequencing. Only the Syrian cohort was screened for all 22 variants (20 diseases). The Iranian cohort was screened for only 20 of the 22 variants (18 diseases) because the amplicon sequencing assays for COL6A2:c.1402C>T and FXN:GAA expansion genotyping were not available at the time of the Iranian cohort screen. Table 3 shows an increased frequency of category 1 variant frequency in Syrian Jews compared to Iranian and Ashkenazi Jews (not including the two variants for which no Iranian data was generated, and not including GJB2:c.167del which is much more common in Ashkenazis). On the other hand, category 2 and 4 variant frequencies were higher in Iranians than in Syrians and Ashkenazis (Table 3). These results were expected given the variant selection criteria described above. However, notably the category 3 inclusion body myopathy‐associated GNE:c.2228T>C variant is present in both Iranians (3.81%) and Syrians (0.71%) and much less so in Ashkenazis (0.09%; Table 3) which might explain why Dor Yeshorim previously identified disease‐affected children in both Iranian and Syrian ethnic groups (unlike category 1 variants which were primarily identified in disease affected children of Syrian Jewish ancestry, and unlike category 2 variants for which affected children were exclusively Iranian Jewish). Regarding the pan‐ethnic category 5 CFTR:c.1521_1523del variant, Table 3 shows similar frequency in Syrian (0.56%) and Iranian (0.47%) groups but much higher frequency in Ashkenazis (1.16%). Thus, there are shared alleles between Syrian, Iranian, and Ashkenazi Jews as well as some alleles unique to each group. In addition, we also note that 8.2% of the 3401 mixed Syrians were each carriers for at least one of the pathogenic variants in Table 3.

**TABLE 3:** Carrier frequencies in mixed Syrian, Iranian, and Ashkenazi Jewish cohorts

Variant Name	Variant category	Syrian Jewish carrier frequency (no. carriers/*n*)	Syrian Jewish carrier frequency %	Iranian Jewish carrier frequency (no. carriers/*n*)	Iranian Jewish carrier frequency %	Ashkenazi Jewish carrier frequency (no. carriers/*n*)	Ashkenazi Jewish carrier frequency %
COL6A2:c.1402C>T	1	56/3401	1.65	ND	ND	29/10125	0.29
GJB2:c.167del	1	41/3401	1.21	3/1112	0.27	1078/41414	2.60
FXN:GAA expansion	1	19/3401	0.56	ND	ND	26/9208	0.28
CYP11B1:c.992C>T	1	9/3401	0.26	2/3010	0.07	6/60345	0.01
NDUFS4:c.355G>C	1	6/3401	0.18	2/3132	0.06	16/67454	0.02
VAC14:c.2005G>T	1	5/3401	0.15	0/1087	0.00	0/39212	0.00
ESCO2:c.1674‐2A>G	1	4/3401	0.12	0/3010	0.00	7/61630	0.01
CNGB3:c.467C>T	1	4/3401	0.12	3/2904	0.10	37/59336	0.06
BLM:c.98+1G>T	1	1/3401	0.03	0/1088	0.00	1/39219	0.00
ARSA:c.854+3A>G	1	1/3401	0.03	0/3010	0.00	1/61747	0.00
ARSA:c.449C>T	1	1/3401	0.03	0/3236	0.00	8/67953	0.01
AGXT:c.731T>C	1	1/3401	0.03	0/347	0.00	2/27885	0.01
CFTR:c.254G>A	1	0/3401	0.00	1/3576	0.03	4/117781	0.00
GPT2:c.159C>G	1	0/3401	0.00	0/3007	0.00	1/61586	0.00
DSE:c.387delC	1	0/3401	0.00	0/342	0.00	0/27799	0.00
AIRE:c.254A>G	2	9/3401	0.26	55/3761	1.46	18/73327	0.02
LIPA:c.260G>T	2	1/3401	0.03	15/1435	1.05	8/40987	0.02
GNE:c.2228T>C	3	24/3401	0.71	120/3147	3.81	61/67615	0.09
TYMP:c.433G>A	4	4/3401	0.12	34/3747	0.91	10/73272	0.01
USH2A:c.236_239dup	4	2/3401	0.06	18/3588	0.50	10/65282	0.02
CNGA3:c.1585G>A	4	1/3401	0.03	11/3761	0.29	9/73408	0.01
CFTR:c.1521_1523del	5	19/3401	0.56	25/5279	0.47	4303/370954	1.16

Note

Data is shown for unrelated individuals with at least one Syrian Jewish grandparent or at least one Iranian Jewish grandparent or at least one Ashkenazi Jewish grandparent, as indicated.

Abbreviations: N/A, not applicable; ND, no data.

## 4 DISCUSSION

We report carrier frequencies for pathogenic variants underlying 25 different autosomal recessive diseases in Syrian Jewish individuals. For 3 of the variants (AIRE:c.254A>G [autoimmune polyendocrinopathy], LIPA:c.260G>T [Wolman’s disease], and GNE:c.2228T>C [inclusion body myopathy (HIBM)]), carrier frequency was assayed previously in Mizrahi Jews and found to be similar to the frequencies reported here (Kaback et al., 2010; The Forward Staff, 2014). Most importantly, we show that at least one pathogenic variant was present in 438 Syrian Jews with four Syrian grandparents, and 20 of these variants (underlying 17 different conditions) are present at a population frequency (>0.6%) for which premarital screening would be recommended. In terms of disease severity and allele frequency, the American College of Medical Genetics (ACMG) guidelines recommend that most of these 20 disease‐causing variants should be tested in the Syrian Jewish population (Grody et al., 2013). These criteria include (1) the genetic disorder being tested is severe such that most at‐risk couples would pursue prenatal diagnosis if given the option; (2) a clear association exists between the assayed variant and a severe early onset genetic disorder; and (3) relatively high carrier frequency is present in the tested population such that carrier screening would help to reduce disease incidence. Although these criteria have been met for most of the variants in Tables 1 and 3, for many of these variants (notably COL6A2:c.1402C>T [underlying Ullrich congenital muscular dystrophy type 1] which is present in 1.65% of Syrian Jews) carrier frequency had not been described previously in Syrian Jews.

Testing a small cohort of 100% Syrian Jews (descended from 4 Syrian Jewish grandparents) identified 33 disease‐causing variants underlying 25 autosomal recessive conditions. Sixteen of these variants (underlying 13 autosomal recessive conditions) are also present in Ashkenazi Jews. In addition, 22 variants (20 of the 25 conditions) were tested in a larger mixed Syrian Jewish cohort. Predictably, several pathogenic variants were identified with relatively high carrier frequency in both 100% Syrian Jewish and mixed Syrian Jewish cohorts. Thus, the findings of this study suggest that carrier screening could be clinically useful. Based upon our calculated carrier frequencies, 0.82% (82 of 10,000) of Syrian Jewish couples with 4 Syrian grandparents would both be carriers for disease‐causing variants in the same gene for one of 33 variants in 25 genes listed in Table 1. Likewise, carrier frequencies in the mixed Syrian Jewish group show that 0.06% of mixed Syrian Jewish couples (6 out of every 10,000) would both be carriers for disease‐causing variants for one of the 22 variants in 20 genes in Table 3.

All Jewish groups carry pathogenic disease‐causing recessive variants, and Syrian Jews are no exception (Zlotogora, 2014). Moreover, it is becoming increasingly clear that many pathogenic variants previously thought to be exclusive to one Jewish group are present in other Jewish groups. Therefore, these data suggest that more expanded carrier screening panels should be used to address premarital screening in all Jewish groups because what was previously thought to be exclusively an Ashkenazi variant, for example, CFTR:c.3846G>A, is also present in Syrian Jews. Therefore, it is likely that the carrier screening panel will continue to expand as new disease alleles are identified. In addition, it appears that ethnic‐specific screens are less effective, especially given that expanding the number of tested alleles adds minimal cost. Rather a comprehensive pan‐Jewish panel should include variants identified in Jews of all ancestries, especially now that admixture of Jews of different ancestries occurs more frequently.

Based on the findings in this study, Dor Yeshorim has initiated new educational programming to raise awareness about premarital reproductive planning within Syrian Jewish communities. The disease risk within this group is apparent, and carrier screening is recommended by ACOG for all patients.

The relatively short list of variants assessed and reported in this study was not designed to be exhaustive for all conditions that may be recommended for Syrian Jewish carrier screening. We acknowledge that there are other disease‐causing recessive disorders in Syrians that were not included in this study and, accordingly, we list those that are presently known to us based on literature and the Israeli National Genetic Database (“The Israeli National Genetic Database,” n.d.) in Table S1. Notably, we also did not report the Syrian Jewish carrier frequency for *SMN1* (MIM#: 600354) for spinal muscular atrophy (SMA) even though it is the most common autosomal recessive disease across worldwide populations (Verhaart et al., 2017). This is not to imply that SMA and other recessive diseases are not common in Syrian or Iranian Jews as well. On the contrary, we predict that numerous disease‐causing alleles not described in this report are likely to be present in Syrian Jews with sufficiently high frequency and severity to justify inclusion in premarital screening. However, the goal of this study was not to establish definitive carrier frequencies for all pathogenic variants in Syrian Jews. Instead, we chose a diverse group of variants which were obtained primarily through our work with Syrian families that were affected by genetic diseases for which they had no prior awareness. As we identify these recessive conditions in families in the community, it will be important to increase awareness and to have the infrastructure to disseminate these findings to premarital couples who are likely to be carriers for one or more conditions given the degree of shared ancestry in these small populations.

## CONFLICTS OF INTEREST

The authors declare no conflicts of interest.

## AUTHOR CONTRIBUTIONS

Conceptualization: D.A.Z., S.Y.S., Y.H., J.E.; Data curation: D.A.Z., C.L., Y.H., T.W.; Formal Analysis: D.A.Z., C.L., Y.H.; Investigation: D.A.Z., W.K.C., C.L., S.Y.S, Y.H., T.W., A.E., J.E.; Methodology: D.A.Z., R.B., Y.K., H.M., R.B.; Resources: A.E., J.E.; Supervision: D.A.Z., A.E., J.E.; Validation: D.A.Z., W.K.C., C.L., R.B., Y.K., H.M., R.B., Y.H., T.W.; Writing – original draft: D.A.Z.; Writing—review & editing: D.A.Z., W.K.C., S.Y.S., Y.H., A.E., J.E.; Approval of final version of manuscript: All authors.

## EDITORIAL POLICIES AND ETHICAL CONSIDERATIONS

Ethical approval for this study was obtained from the Dor Yeshorim institutional review board according to guidelines of the Declaration of Helsinki. DNA samples were obtained with written informed consent from self‐identified Syrian, Iranian, or Ashkenazi Jewish individuals enrolled in the Dor Yeshorim carrier testing program.

## Supporting information

Table S1

## Data Availability

The source data for this study is available upon reasonable request.
